# Exploitation of Other Social Amoebae by *Dictyostelium caveatum*

**DOI:** 10.1371/journal.pone.0000212

**Published:** 2007-02-14

**Authors:** Clément Nizak, Robert J. Fitzhenry, Richard H. Kessin

**Affiliations:** 1 Living Matter Laboratory, Center for Physics and Biology, Rockefeller University, New York, New York, United States of America; 2 Department of Anatomy and Cell Biology, Columbia University, New York, New York, United States of America; University of Minnesota, United States of America

## Abstract

*Dictyostelium* amoebae faced with starvation trigger a developmental program during which many cells aggregate and form fruiting bodies that consist of a ball of spores held aloft by a thin stalk. This developmental strategy is open to several forms of exploitation, including the remarkable case of *Dictyostelium caveatum*, which, even when it constitutes 1/10^3^ of the cells in an aggregate, can inhibit the development of the host and eventually devour it. We show that it accomplishes this feat by inhibiting a region of cells, called the tip, which organizes the development of the aggregate into a fruiting body. We use live-cell microscopy to define the *D. caveatum* developmental cycle and to show that *D. caveatum* amoebae have the capacity to ingest amoebae of other Dictyostelid species, but do not attack each other. The block in development induced by *D. caveatum* does not affect the expression of specific markers of prespore cell or prestalk cell differentiation, but does stop the coordinated cell movement leading to tip formation. The inhibition mechanism involves the constitutive secretion of a small molecule by *D. caveatum* and is reversible. Four Dictyostelid species were inhibited in their development, while *D. caveatum* is not inhibited by its own compound(s). *D. caveatum* has evolved a predation strategy to exploit other members of its genus, including mechanisms of developmental inhibition and specific phagocytosis.

## Introduction

The social amoebae ingest bacteria by phagocytosis and then, when the bacteria are consumed, aggregate to form a fruiting body with spores and a stalk [1]. The unusual features of *D. caveatum*, first described by Waddell and colleagues nearly 20 years ago, are displayed when it is mixed with other species of social amoebae [2]. Even when only a few *D. caveatum* amoebae are present in an aggregate of 10^4^ *D. discoideum* amoebae, *D. caveatum* emerges as the only species present. In a few days, all of the *D. discoideum* amoebae are ingested and *D. caveatum* fruiting bodies emerge from each *D. discoideum* aggregate. *D. caveatum* has the same effect on other species of social amoeba.

This predatory behavior of *D. caveatum* has not been observed among other Dictyostelid species: when starving amoebae of different Dictyostelid species are mixed, they sort out and form their respective fruiting bodies [3], [4]. While predation between related species is common, what is remarkable in the case of *D. caveatum* predation of other Dictyostelids is that, so far, only one species seems to be an efficient and specific predator of other members of its genus, of which nearly a hundred species have been identified [5].

Waddell and colleagues mixed cells of *D. caveatum* and other Dictyostelids, counted them over time, and concluded that *D. caveatum* cells divide, while the other cell type decreases. Electron microscopic observation showed prey amoebae engulfed in small pieces, and therefore Waddell proposed that *D. caveatum* feeds on other amoebae but not on itself and forms fruiting bodies in mixtures with other Dictyostelia [2], [6].

To characterize the predatory mechanisms of *D. caveatum* we observed the direct interactions of *D. caveatum* with *D. discoideum* by live-cell microscopy, in particular to demonstrate, this time directly, phagocytosis of *D. discoideum* by *D. caveatum*. Further, we have identified a system that *D. caveatum* uses to inhibit the development of the prey cells, which has the effect of conserving the biomass of *D. discoideum*, or related species, for the benefit of *D. caveatum*. During *D. discoideum* development, cells aggregate, then differentiate into essentially two cell types and sort out, a majority of prespore cells and a smaller number of prestalk cells, which accumulate in an apical region called the tip. This structure acts as an organizer of the cell aggregate: grafting of exogenous tips onto an aggregate induces the formation of a slug at the site of each tip [7]. *D. caveatum* inhibits the development of Dictyostelid species by preventing tip formation and thus the organization and developmental progression of the prey cell aggregate.

One of many similar cases can be found in the infection of insects or insect larvae by the parasitoid wasp *Ampulex compressa*. This wasp injects its venom into the ganglia of cockroaches, which incapacitates the prey. The wasp thus alters the metabolism of the insect host it inhabits, and uses it as a source of nutrients [8], [9]. *D. caveatum*, a eukaryotic microorganism, can inhibit the development of its prey, ingest it and produce its own fruiting bodies from within the aggregates of the prey. However, unlike the case of the wasp, *D. caveatum* is closely related to its prey.

## Results

### Direct observation of phagocytosis

First, we confirmed the observations of Waddell *et al.* in mixtures of *D. caveatum* cells with *D. discoideum* cells placed in starving conditions. We found that only *D. caveatum* forms fruiting bodies when it constitutes anywhere from 1/10^3^ to 10^3^/1 of the cells in the initial mixture. *D. discoideum* fruiting bodies formed only when the ratio was below 1/10^4^ or when *D. caveatum* was added (even in vast excess) to developing *D. discoideum* cells that had already formed slugs (data not shown). *D. caveatum* develops more robustly when grown on *D. discoideum* than on bacterial lawns (data not shown) in our culture conditions (which may however not be optimal)*.*

**Figure 1:**
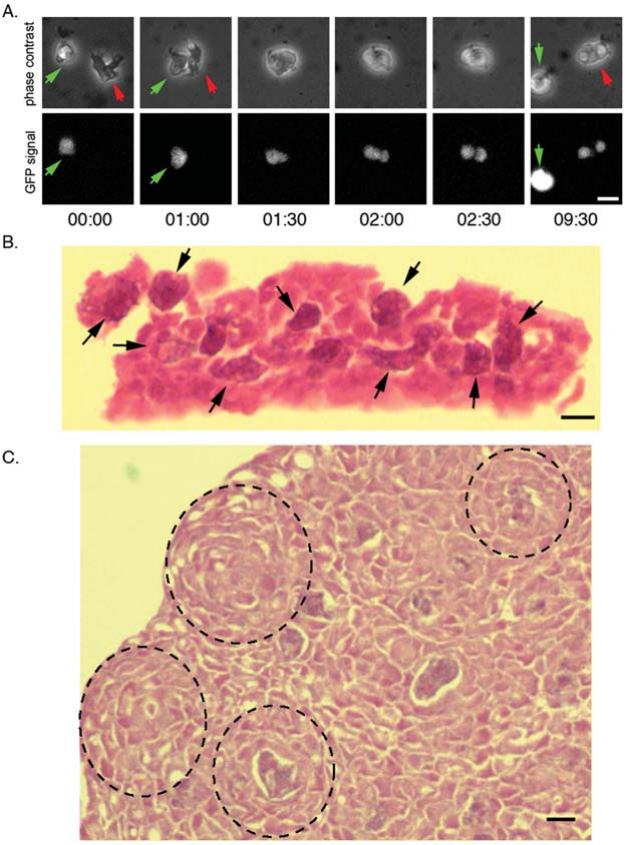
The phagocytosis of *D. discoideum* by *D. caveatum*. A. Time series of phagocytosis. A red-labeled *D. caveatum* amoeba (red arrow) phagocytoses a GFP-expressing *D. discoideum* amoeba (green arrow). Engulfment is complete within 2 minutes following contact. The prey cell is fragmented into several phagosomes within the same time frame (see GFP-positive fragments at 2.5 minutes following contact). After less than 10 minutes, phagocytosis is complete and the *D. caveatum* amoeba resumes migration until it comes into contact with another prey cell. Time is in minutes and seconds (MM:SS, see Movie S1A and S1B). Bar = 10 µm. B. Histological section of *D. caveatum* amoebae in an aggregate where the prey cells were initially at 1 *D. caveatum* for 1000 prey cells. *D. caveatum* amoebae are presumably the large darker cells showing a cytoplasmic basophilic straining (arrows), containing material resembling the majority of cells in the aggregate (fragments of *D. discoideum* cells). This section was prepared 16 hours after the beginning of development. Bar = 10 µm. C. Histological section of an aggregate in which nearly all *D. discoideum* amoebae have been consumed and only *D. caveatum* cells remain. These cells are packed in dense aggregates, and a few of them still contain remnants of *D. discoideum* amoebae. Dotted lines have been drawn around foci of aggregating *D. caveatum* within the cellular mass. This section corresponds to 36 hours after the beginning of development. Bar = 10 µm.

Based on transmission electron microscopic analysis of *D. caveatum*/*D. discoideum* mixtures showing cells containing ingested amoebae, Waddell proposed that *D. caveatum* feeds on other species, but not itself. This argument was reinforced by the observation of fluorescent compartments in *D. caveatum* amoebae incubated with fluorescamine-stained *D. discoideum* [6]. We examined this prediction by observing single living cells of *D. caveatum* and *D. discoideum* labeled with different fluorophores (Figure 1A, Movies S1A and S1B). Time-lapse microscopy revealed that *D. caveatum* cells are very motile, have a distinct morphology, and perform rapid phagocytosis of *D. discoideum*. From the time of cell-cell contact to ingestion, only 2–3 minutes are required in comparison to 30–45 minutes that has been reported for macrophages phagocytosing particles of their own size [10]. *D. caveatum* cells extend pseudopods of about their size (5–10 microns) in 10–30 seconds, surround their prey, engulf it, and reduce it to several phagosomes (containing GFP in the case of GFP-expressing *D. discoideum* prey cells). *D. caveatum* cells phagocytose live cells. We have not observed that they ingest debris or dead cells. When the prey cell is large, *D. caveatum* cells leave a non-ingested piece of live cell behind. When two *D. caveatum* cells come into contact, they do not surround each other; rather they migrate in different directions (see Movie S1C).

This phagocytosis process occurs in *D. caveatum*/*D. discoideum* mixed aggregates. This idea is supported by histological sections of such aggregates (Figure 1B) showing dark cells, presumably *D. caveatum* cells, filled with phagosomes containing material resembling the majority of cells in the aggregates, the *D. discoideum* cells. After the *D. discoideum* have been consumed and only *D. caveatum* remain, the *D. caveatum* cells form aggregates. Some of these cells still contain remnants of *D. discoideum* amoebae but most of them have processed their phagosomes and changed their morphology (Figure 1C): cells have a more homogenous subcellular organization and are densely packed in aggregates. Foci of circularly arranged cells can be seen within the mass, from which *D. caveatum* aggregates arise. We have not used a specific antibody in this experiment, but we infer from the cell numbers and the distinct phagocytic properties of the minority cells that the large phagosome-filled cells are *D. caveatum*.

We tested a number of species according to the same protocol as Figure 1A to determine whether they are consumed by *D. caveatum.* We found that *D. aureostipes, D. fasciculatum, D. mucoroides, D. rosarium* and *P. pallidum* are also preyed on by *D. caveatum* (see Movie S1D). *D. caveatum* phagocytoses all Dictyostelid species tested so far. It was not possible to test all known species, but we chose at least one representative species of each group defined by a phylogenetic tree constructed by Schaap and colleagues [5]. We also tested the effect of *D. caveatum* on *Acrasis rosea*, which, though it is amoeboid and has an aggregative developmental cycle, is unrelated to the Dictyostelia [11], [12]. Its amoebae are also ingested by *D. caveatum* (not shown).

### *D. caveatum* inhibits tip formation by blocking coordinated cell movement but not differentiation

Even with such an efficient phagocytic predation system, *D. caveatum* would not be able to consume a cell aggregate of 10^4^–10^5^ *D. discoideum* amoebae if it were initially present as one or a few cells. During the 12–16 hours it takes for the *D. discoideum* amoebae to aggregate, only 3 or 4 cell divisions of *D. caveatum* could occur. We therefore examined the dynamics of predation of large populations.

Time-course experiments and phase contrast time-lapse microscopy acquisition yielded more details of *D. caveatum* predation. *D. caveatum*/*D. discoideum* mixtures in which *D. caveatum* is in excess are indistinguishable from pure *D. caveatum* preparations: only *D. caveatum* fruiting bodies form and after 48 hours no trace of *D. discoideum* remains. When *D. discoideum* is present in an excess of 10^3^ to 10^4^ fold, aggregation of *D. discoideum* cells appears normal, with the same kinetics as pure *D. discoideum* preparations, leading to aggregate formation 10–12 hours after the food was removed. However, unlike the control mixtures, the aggregates do not produce tips, which in *D. discoideum* normally form on top of the mounds and act to organize further development. It is from these frozen aggregates that *D. caveatum* fruiting bodies emerge after about 36 hours. When the ratio is *D. caveatum*/*D. discoideum* = 1/10^2^, aggregates are arrested at an even earlier stage and become frozen at the loose aggregate stage. In this case, after 16–18 hours of development, it is likely that about 10% of the aggregate would be highly phagocytic *D. caveatum* and the *D. discoideum* amoebae may be so damaged that they cannot progress to the tight aggregate stage.

**Figure 2:**
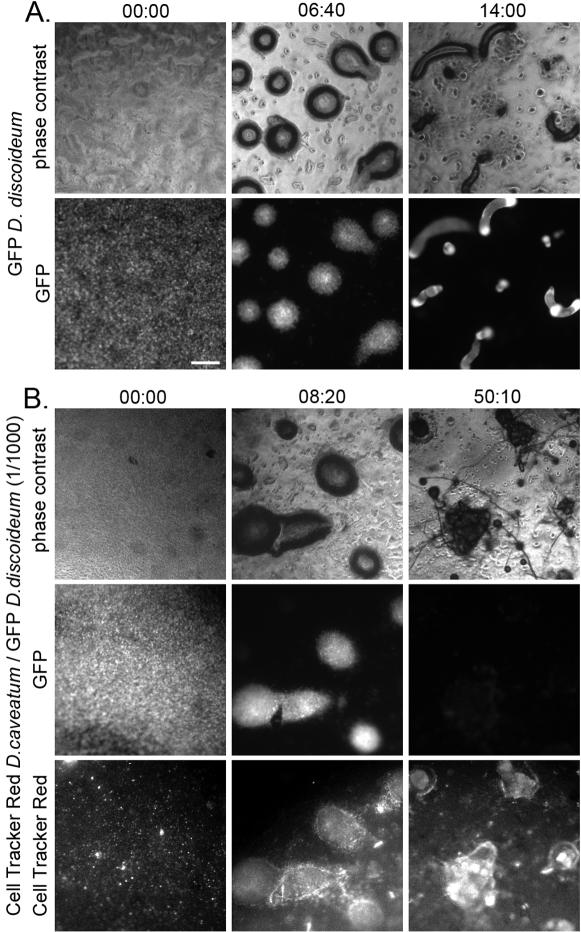
Time-lapse microscopy of the development of *D. caveatum*/*D. discoideum* mixtures. Cells of control GFP-expressing *D. discoideum* populations (A) and GFP-expressing *D. discoideum* populations containing 1/10^3^ Cell Tracker Red-labeled *D. caveatum* (B) were allowed to develop for 36 hours and continuously observed by time-lapse video microscopy. A. The control *D. discoideum* cultures undergo development from aggregation to fruiting (collective circular motion within aggregates is observed; see Movie S2A). Three stages are shown here: beginning of development (before aggregation), the tight aggregate stage, and the slug stage. Time is in HH:MM and 00:00 corresponds to the beginning of recording, during the initiation of aggregation. Bar = 200 µm. B. Aggregates infected with *D. caveatum* are blocked at the aggregate stage and no collective circular motion is observed (see Movie S2B). Finally *D. caveatum* amoebae emerge as slugs and fruiting bodies when all of *D. discoideum* amoebae are consumed (and the GFP signal disappears).

Mixtures of cells of both species were observed by time-lapse fluorescence microscopy. Control GFP-expressing *D. discoideum* cells aggregate and, as has been described by C. Weijer and others [13]–[15], there is a collective rotational motion of amoebae within the aggregate before and during the formation of the tip (see Movie S2A). These aggregates then progress to form slugs (Figure 2A). In contrast, *D. discoideum* aggregates that have been infected with 1/10^3^ Cell Tracker Red-labeled *D. caveatum* amoebae do not perform this collective circular motion (see Movie S2B). In some aggregates, rotational motion is initially observed but it stops (this is even more common at 1/10^4^). The GFP signal decreases as *D. discoideum* amoebae are consumed (Figure 2B). When the prey *D. discoideum* population is exhausted, red-labeled *D. caveatum* aggregate, form very small slugs and then fruiting bodies (Figure 2B). A number of slugs and fruiting bodies of *D. caveatum* emerge from a former *D. discoideum* aggregate. Only a few red-labeled *D. caveatum* cells are visible initially in each *D. discoideum* aggregate. However, there are sufficient predator cells to overwhelm the prey population.

Our results suggest that, at a ratio of 1/10^3^, *D. caveatum* induces a block in the development of *D. discoideum* at the tight aggregate stage: the collective rotational motion and morphogenesis of the tip are inhibited. *D. discoideum* amoebae in these frozen aggregates are then consumed by *D. caveatum*, and subsequently *D. caveatum* turns off its feeding program and triggers its own developmental program, with a consequent change in cellular morphology.

**Figure 3:**
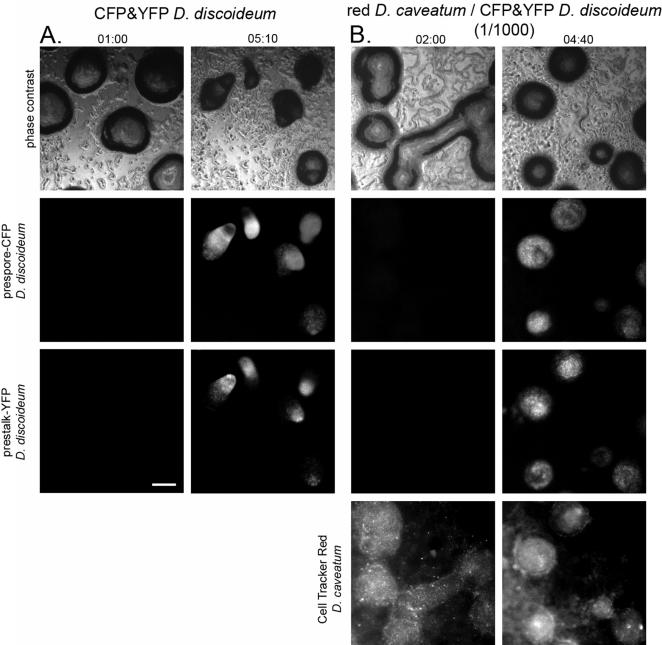
In mixtures of 1/10^3^ *D. caveatum*/*D. discoideum,* aggregation is blocked but cell-type differentiation occurs. Time in HH:MM. 00:00 corresponds to the beginning of recording, during the initiation of aggregation. Bar = 200 µm. A. In control developing *D. discoideum* populations, amoebae start to express CFP and YFP under the control of pre-spore or pre-stalk promoters after aggregation but before tip formation. The collective rotational motion of amoebae in aggregates is observed (see Movie S3A). These markers are subsequently expressed in the pre-spore or pre-stalk regions of slugs and finally the stalk and spores of fruiting bodies (see Movie S3A). Two stages are shown here: late aggregation phase, before the fluorescent reporters are expressed; and tip formation, when the markers are already fully expressed and cells have sorted out to different regions of these aggregates. B. In 1/10^3^ *D. caveatum*-infected *D. discoideum* populations, these markers are also expressed after aggregation. However there is no collective rotational motion (see Movie S3B) and no tip formation. These markers persist until all *D. discoideum* amoebae are ingested and starving *D. caveatum* triggers its own development program. Here we show the late aggregation phase, before the fluorescent reporters are expressed; and the blocked aggregate phase, when markers are expressed but no tips form on aggregates.

We next asked whether the inhibitory effect of *D. caveatum* was due to inhibition of cell-type differentiation. We reasoned that a block in prestalk cell differentiation would create a block at the aggregative stage with the absence of tip formation, as observed. Accordingly, we created a set of plasmids that use prespore (*pspA*) and prestalk (*ecmA*) cell specific promoters fused to CFP and YFP respectively to examine cell-type differentiation in control *D. discoideum* populations and *D. caveatum*/*D. discoideum* mixed cultures. *D. discoideum* transformants carrying these constructs express them only after aggregation, when cell-type differentiation begins. The CFP and YFP signals can be detected in the prespore and prestalk regions respectively of tipped aggregates and slugs (Figure 3A, Movie S3A). Under conditions in which 1/10^3^ of the cells in a *D. discoideum* aggregate are *D. caveatum, D. discoideum* cells reach the aggregate stage and express fluorescent proteins under the control of prestalk or prespore specific promoters. These reporter genes are expressed at similar levels in the absence or presence of *D. caveatum*, as assessed by comparing fluorescence intensity levels in Figure 3A and 3B. As in the earlier experiment, the collective circular motion of the cells is blocked, even though cell movement still occurs. (Figure 3B and see Movie S3B). Therefore, the presence of *D. caveatum* in *D. discoideum* aggregates does not prevent the expression of genes under the control of developmental promoters, either prestalk or prespore, even though morphogenesis (tip formation) is blocked.

### A small compound inhibits development

**Figure 4:**
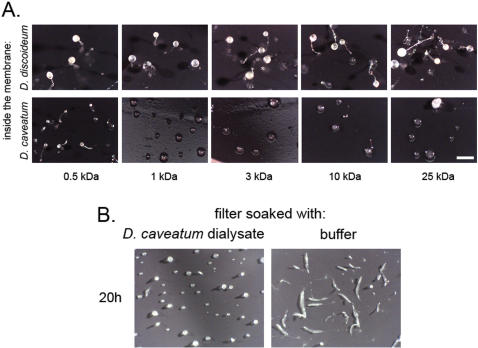
A chemically induced block to development. A. Dialysis membranes of various pore sizes were filled with starving amoebae of *D. discoideum* (control) or *D. caveatum* at 10^7^/mL. Millipore filters, on which test populations of *D. discoideum* had been deposited, were placed on top of the dialysis membranes. The two populations were in chemical but not physical contact (see Figure 5). After 24 hours of development of the test populations, fruiting was complete in the case of the *D. discoideum*-filled membranes, partial in the case of the *D. caveatum*-filled 500 Da membrane. Development was blocked at the aggregation stage, in the case of *D. caveatum*-filled membranes of size greater than 1 kDa. Bar = 500 µm. A dialysate of a *D. caveatum*-filled membrane also inhibits development of *D. discoideum*. A dialysis membrane filled with *D. caveatum* was dialysed against 1.5 mL of SorC buffer for 24 hours. Test populations of *D. discoideum* on filters were then incubated with this dialysate or SorC (control). At 20 hours of development, the dialysate had inhibited *D. discoideum* development at the aggregate stage while control *D. discoideum* had reached the slug stage. The inhibition was a 4–6 hour lag at aggregation stage. Bar = 500 µm.

The developmental inhibition at the tight aggregate stage could be mediated through direct cell-to-cell contact, or by secretion of an inhibitory compound. We tested these two possibilities by observing the development of *D. discoideum* on a filter that was placed onto a semi-permeable dialysis membrane containing starving *D. caveatum* cells, *D. discoideum* cells, or a mixture of both. While *D. discoideum* development was normal when *D. discoideum* cells were in the dialysis membrane, it was inhibited when *D. caveatum* cells were in the membrane, either alone or in mixture with *D. discoideum*. Inhibition consisted in a complete block at the aggregation stage. By using dialysis membranes with different pore sizes, we estimated the size of the inhibitor. Complete inhibition occurred in all cases except when the pore size was less than 1 kDa (inhibition is only partial at 500 Da), as shown in Figure 4A. This result showed that cell-cell contact was not required for the inhibition to occur at its maximum level (complete block), and that a small molecule or molecules (<1 kDa) exchanged through the membrane were responsible for inhibition.

We isolated molecules of less than 1kDa that are secreted by starving *D. caveatum* by dialyzing a *D. caveatum*-containing dialysis membrane against buffer. When *D. discoideum* cells were developed on filters that were soaked with this dialysate, development was delayed at the aggregate stage for 4–6 hours compared to the control buffer-soaked filter (Figure 4B). Both the test filters and control filters went on to form fruiting bodies after 48 hours (not shown). This result implies that *D. caveatum* secretes a development inhibitor in the absence of prey cells. It also indicates that the inhibition is stronger when cells are in chemical contact through a dialysis membrane perhaps because the inhibitor is unstable and needs to be continuously secreted by *D. caveatum* to sustain inhibition of *D. discoideum* development.

We performed a preliminary chemical characterization of the inhibitory compound(s). Inhibitory activity was found in the aqueous phase but not in the organic phase after extraction with various organic solvents. It was also present in one fraction of a separation on a negatively charged ion exchange column (absent from the flow through) and the flow through of a positively charged ion exchange column (absent from all eluted fractions). Activity could be preserved, or even restored, with the reducing agent DTT. These preliminary results indicate that the inhibitory compound is a small hydrophilic molecule that is negatively charged and sensitive to oxidation.

### Reversibility and spectrum of the developmental inhibition

The inhibition process is not a simple killing mechanism. This developmental block is reversible: Test populations of *D. discoideum* placed onto filters that had been incubated for 24 hours on top of *D. caveatum*-filled dialysis membranes were transferred to buffer. Approximately 75% of *D. discoideum* aggregates that were blocked in development after the first incubation period progressed during the subsequent incubation period to complete the full development cycle, resulting in fruiting body formation (Table 1).

**Table 1:** Reversibility of the inhibition.

	% of structures at the aggregate stage	
Primary incubation	buffer	*D. caveatum*
24hrs	19 (9)	98 (4)
Secondary incubation with buffer		
24 hrs	15 (11)	34 (10)
48 hrs	8 (4)	26 (9)
72 hrs	8 (4)	26 (10)

**Figure 5:**
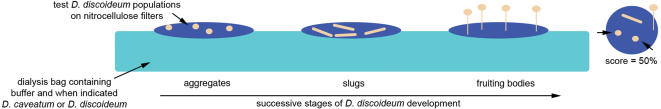
Diagram accompanying Tables 1, 2 and S1.

Developing *D. discoideum* cells were incubated on filters on top of membranes containing *D. caveatum* or buffer (control) for 24 hours. Filters were then transferred to buffer-filled membranes and scored after 24, 48 and 72 hours. Each score represents the number of aggregates as a percentage of the total number of developmental stages observed at the time indicated (average (standard deviation)), as illustrated in Figure 5. The development inhibition is reversible: ∼75% of the *D. caveatum*-inhibited *D. discoideum* aggregates complete development to fruiting when rescued on SorC buffer for 72 hours.

We asked whether this developmental inhibition occurs for other prey species. We used test populations of starving cells of *D. aureostipes, D. fasciculatum, D. mucoroides* and *D. rosarium* deposited onto a filter on top of a buffer-filled dialysis membrane (control) or *D. caveatum*-filled dialysis membrane. In all cases, the development of the test population was delayed at the aggregate stage after 24 hours (Table 2). Therefore *D. caveatum* inhibits the development of several species of its own genus, but not itself. The inhibition employs a small diffusible compound, although not necessarily the same one for each species. This inhibition at the aggregate stage is also visible in 1/10^3^ mixtures of *D. caveatum* with each of these species: as for *D. caveatum*/*D. discoideum* mixtures, development is visibly “frozen” at the aggregate stage compared to controls after 24 hours (Table S1). *D. caveatum* does not inhibit the development of *Acrasis rosea*, an unrelated organism with a similar life cycle, although it will eat it (data not shown).

**Table 2:** Range and specificity of inhibition and phagocytosis

	% of structures at the aggregate stage		Phagocytosis of prey cells
	buffer	*D. caveatum*	by *D. caveatum*
*D.* *aureostipes*	37 (28)	83 (30)	+
D. fasciculatum	24 (18)	70 (26)	+
D. mucoroides	14 (15)	100 (0)	+
D. rosarium	4 (7)	97 (6)	+

Four additional Dictyostelid species were tested according to the same protocol as in Figure 4A. The table indicates the number of aggregates for each species as a percentage of the total number of developmental stages observed on filters after 24 hours of incubation (average (standard deviation)), as illustrated in Figure 5. In the controls, development at 24 h has passed the aggregate stage, while in the presence of *D. caveatum* (10^8^ cells/mL inside the dialysis membrane) development is blocked at this stage (compare first two columns). The ability of *D. caveatum* to phagocytose ameobae of these species, as assessed by live cell microscopy, is also indicated: all these Dictyostelid species are ingested by *D. caveatum* in the same manner as *D. discoideum* (Movie S1D).

## Discussion

*D. caveatum* acts as a predator for all tested Dictyostelid species. This mechanism results in the inhibition of Dictyostelia development at a stage at which *D. caveatum* can still feed on its prey.

We used live-cell microscopy to define the predatory mechanisms of *D. caveatum* and confirmed that non-self specific phagocytosis and self-avoidance are the basis of its predation. Our observations indicate that *D. caveatum* ingests living cells of species that belong to different groups of an evolutionary tree [5], as well as the unrelated developing amoeba *Acrasis rosea*. D. Waddell characterized mutants of *D. caveatum* that seem to have lost their capacity for self-avoidance and were therefore labeled cannibalistic [16], [17]. It will be interesting to observe such mutants by live-cell microscopy to confirm the phenotype and examine the molecular mechanisms of non-self specific phagocytosis in *D. caveatum*. Another hint at these mechanisms may come from the study of the giant zygote of the *D. discoideum* sexual development, which has been observed to perform the exact inverse task: specifically ingesting cells of its own species only [18].

We identified another mechanism that provides *D. caveatum* with a larger number of prey cells and hence nutrients: *D. caveatum* blocks the development of species within its genus, but, remarkably, not its own development. Our results indicate that *D. caveatum* blocks the formation of the tip, the organizer of cell aggregates: *D. caveatum* targets mechanisms by which *D. discoideum* amoebae coordinate their collective movement during development and morphogenesis, without blocking the molecular development program at the single cell level. Parenthetically, this result suggests that it is possible to uncouple differentiation from morphogenesis by targeting the coordination between cells, an idea that has been outlined in previous studies where cells were prevented from accomplishing morphogenesis [19], [20].

To inhibit the development of Dictyostelid species but not its own, *D. caveatum* must employ very specific mechanisms. *D. caveatum* secretes one or several small compound(s) constitutively, even in the absence of prey cells, that induce a reversible block in prey cell development at the tight aggregate stage. The mechanisms by which the inhibitory compound functions and *D. caveatum* escapes developmental inhibition by its own compound remain to be addressed.

Such an efficient predation process might cause the disappearance of most other Dictyostelid species. Yet, two observations indicate that *D. caveatum*'s interactions with its prey are more complex. First, *D. caveatum* was discovered in a cave among several other amoebae species isolates [2]. Second, we observed that while *D. caveatum* fruits efficiently when feeding on other Dictyostelia (perhaps a result of the nutritive advantage of ingesting cells of its own genus), it fruits poorly on bacterial lawns (after several weeks of culture on *Klebsiella pneumoniae*), or surprisingly in *D. caveatum*/*D. discoideum* = 1/1 mixtures (not shown). A detailed analysis of species interactions will be necessary to solve this ecological problem.

*D. caveatum* evolved a non-self phagocytosis process and a non-self development inhibition compound, both efficient and specific, whose mechanisms may have evolved independently. However, *D. caveatum* does not seem to emerge on a separate branch on the Dictyostelia tree [5]. One possibility is that *D. caveatum* has lost some form of phagocytosis inhibition common to all Dictyostelia that prevents other species from eating each other. Nevertheless, *D. caveatum* seems to be a professional phagocyte whose phagocytic prey spectrum extends beyond the Dictyostelia clade, and this property could rather have resulted from a gain of function. In addition, it is also *a priori* difficult to imagine the development inhibition mechanism involving both secretion of a small compound and immunity to this compound to have resulted from a loss of some gene(s). The alternative possibility, that *D. caveatum* evolved two new strategies that make it a predator of its sibling species without diverging from them, is therefore plausible. This constitutes a paradox that deserves study and highlights the surprising role played by *D. caveatum* among the Dictyostelia.

## Materials and Methods

### Growth of strains

*D. discoideum* strains AX3 and DH1 (a uracil auxotroph) were grown in HL5 medium as described [21]. *D. caveatum* has not been adapted for axenic growth and was grown on lawns of *Klebsiella pneumoniae* [17]. Other wild strains and species were grown on lawns of the same bacteria. All wild strains except *D. discoideum* were seeded from stocks of spores onto lawns of bacteria. The appropriate number of spores was used to allow the agar plates to be cleared of *Klebsiella pneumoniae* by 48 hours.

Prior to inhibition and mixing experiments, all strains other than *D. discoideum* were scraped from cleared agar plates and washed free of bacteria by centrifugation at 750×g for 5 min. They were washed at least three times in SorC buffer (Sorensen's buffer containing 50 µM CaCl_2_: 17 mM KH_2_/Na_2_HPO_4_, 50 µM CaCl_2_, pH 6.0).

### Histology

Aggregates of various developmental stages were fixed with formalin, embedded in agar, sectioned at 2 microns, and stained with hematoxylin and eosin.

### Live-cell microscopy

GFP-expressing *D. discoideum* amoebae were obtained by transforming DH1 cells with pTX-GFP and selecting with G418 (geneticin). YFP (citrine) and ECFP (Clontech) were amplified by PCR and cloned into the pTX vector after removing the actin 15 promoter and inserting the *ecmA* or the *pspA* promoters to obtain pTX-*ecmA*-YFP and pTX-*pspA*-CFP. *D. discoideum* amoebae expressing YFP and CFP under the control of development promoters were obtained by transforming DH1 cells with these vectors and selecting with G418. *D. caveatum* cells were labeled in SorC at 10^7^/mL containing 3 µM Cell Tracker Red (Molecular Probes) for one hour at 22°C (and washed 3 times in SorC before and after labeling). Amoebae were imaged with a Leica DMIRB inverted microscope equipped with a CCD camera (Roper Scientific-Princeton Instruments, CT 1300B Cryotiger) and controlled with Image Pro (Media Cybernetics). In the case of phagocytosis experiments, cells were incubated in a SorC or HL5 medium-filled Petri dish, the bottom of which was made out of a glass coverslip (MatTek). Images obtained through a 40× objective were recorded every 5 s–10 s for phase contrast and fluorescence (Chroma filters). For developmental experiments, cells were deposited in a chamber filled with Phytagel (Sigma, 2% in SorC buffer) and imaged through a 5× objective every 45 s for phase contrast and fluorescence. Images were assembled into PhotoJpeg-compressed Quicktime movies using Graphic Converter (Lemkesoft), Image J (http://rsb.info.nih.gov/ij/) and Image Ready (Adobe) on a PowerMac G5 Quad (Apple); 16 bit tiff images were converted to 8 bits and the contrast was linearly adjusted for each movie for optimal visualization. The exact same contrast adjustment was applied to CFP and YFP signals for Movies S3A and S3B (as well as Figures 3A and 3B), and therefore intensity levels can be compared directly between both movies (and figure panels).

### Inhibition through dialysis membranes

Size estimation was accomplished by starving *D. caveatum* 10^7^ cells/mL in SorC in dialysis membranes (Spectrapore) of different pore sizes. A 0.4 µm nitrocellulose Millipore filter was laid on top of the dialysis membrane so that there was a liquid interface between the dialysis membrane and the Millipore filter. Onto this filter, we placed 10^7^ *D. discoideum* amoebae. Control dialysis membranes contained either buffer, buffer contaminated with the bacteria on which the *D. caveatum* amoebae grow, or *D. discoideum* at 10^7^ cells/mL. The inhibition experiments (reversibility, inhibition spectrum) were carried out in triplicate on at least three separate occasions. The dialysate of Figure 4B was obtained by placing the *D. caveatum*-filled dialysis membrane on top of 1.5 mL SorC buffer. The preliminary chemical chromatography experiments were done using sepharose fastflow Q columns (GE Health Sciences).

## Supporting Information

Table S1 *D. caveatum* inhibits the development of several Dictyostelids at the aggregate stage. These mixing experiments involved the addition of *D. caveatum* at the ratio indicated to starving cells of the indicated species. The table indicates the number of aggregates for each species as a percentage of the total number of developmental stages observed on filters after 24 hours of incubation (average (standard deviation)), as illustrated in Figure 5. The presence of *D. caveatum* at 1/10^3^ in mixtures inhibits the development at the aggregate stage. At 1/10^4^ dilutions, the inhibition is no longer visible. The inhibition of *D. rosarium* is less significant at 24 hours than for other species, the predation of *D. caveatum* on this species will be reported elsewhere (R. J. Fitzhenry et al, in preparation).
(0.01 MB PDF)

Movie S1A Movie corresponding to Figure 1A. Cell Tracker Red-labeled *D. caveatum* amoebae (red arrows) and GFP-expressing *D. discoideum* amoebae (green arrows) were incubated in a HL5 medium-filled glass-bottom Petri dish and observed by time-lapse microscopy. *D. caveatum* amoebae ingest live *D. caveatum* amoebae by phagocytosis. Please ignore the artifact present in the red channel.
(8.04 MB MOV)

Movie S1B Repetition of the previous experiment, with non-labeled *D. caveatum*. The same dynamics are observed, the phagocytosis process is not influenced (nor provoked) by the red staining. Note that the first *D. discoideum* cell attacked is not completely ingested, a small remaining piece of cell crawls. This mode of partial phagocytosis was coined nibbling by D. Waddell.
(7.08 MB MOV)

Movie S1C *D. caveatum* amoebae were incubated in a SorC buffer-filled glass-bottom Petri dish and observed by time-lapse microscopy. *D. caveatum* amoebae do not ingest each other, they migrate away from each other immediately upon contact.
(3.16 MB MOV)

Movie S1D Cell Tracker Red-labeled *D. caveatum* amoebae (red arrows) and amoebae of different Dictyostelid species (one species per experiment) were incubated in a SorC buffer-filled glass-bottom Petri dish and observed by time-lapse microscopy. *D. caveatum* cells ingest cells of several Dictyostelid species by phagocytosis.
(9.37 MB MOV)

Movie S2A Movie corresponding to Figure 2A. GFP-expressing *D. discoideum* cells were placed in starving conditions and observed by time-lapse microscopy. During development, a rotation motion of cells within aggregates precedes tip formation.
(6.91 MB MOV)

Movie S2B Movie corresponding to Figure 2B. Cell Tracker Red-labeled *D. caveatum* cells were mixed at 1/10^3^ with GFP-expressing *D. discoideum* cells and placed in the same conditions as in Movie S2A. As aggregates form, some *D. caveatum* cells co-aggregate with *D. discoideum* cells. After aggregation of prey *D. discoideum* cells, no rotation motion is observed within aggregates, and no tip formation occurs. The GFP signal progressively decreases as *D. discoideum* cells are consumed. Red-labeled *D. caveatum* cells finally trigger their development and form slugs and fruiting bodies. Several *D. caveatum* fruiting bodies emerge from an initial *D. discoideum* aggregate.
(7.77 MB MOV)

Movie S3A Movie corresponding to Figure 3A. *D. discoideum* cells bearing plasmids allowing them to express cyan and yellow fluorescent proteins under the control of respectively pre-spore and pre-stalk promoters were mixed together and placed in starving conditions. At the beginning of development, no CFP or YFP signal is observed, until differentiation starts after aggregation. The rotation motion of cells within aggregates is visible (as in Movie S2A), tips form, then slugs and fruiting bodies. Cells expressing CFP are present in the pre-spore region of slugs, cells expressing YFP are enriched in the pre-stalk region.
(8.58 MB MOV)

Movie S3B Movie corresponding to Figure 3B. Cell Tracker Red-labeled *D. caveatum* cells were mixed at 1/10^3^ with *D. discoideum* cells bearing the developmentally regulated CFP and YFP plasmids and placed in the same conditions as in Movie S3A. As aggregates form, some *D. caveatum* cells co-aggregate with *D. discoideum* cells. After aggregation, differentiation starts (both pre-spore and pre-stalk promoters are active). In most aggregates, no rotation motion is observed. In some aggregates there is initially a rotation motion that finally stops. No tip formation is observed in any of the aggregates. CFP and YFP signals decrease as *D. discoideum* cells are consumed. *D. caveatum* triggers then its development and forms slugs and fruiting bodies, several of them emerging from an initial *D. discoideum* aggregate.
(8.17 MB MOV)
