# Supplementary material for: Exploitation of Other Social Amoebae by Dictyostelium caveatum
Source: PLoS One. 2007 Feb 14;2(2):e212. doi: 10.1371/journal.pone.0000212 (PMC1790701; doi:10.1371/journal.pone.0000212)
Supplement: Table S1 — D. caveatum inhibits the development of several Dictyostelids at the aggregate stage. These mixing experiments involved the addition of D. caveatum at the ratio indicated to starving cells of the indicated species. The table indicates the number of aggregates for each species as a percentage of the total number of developmental stages observed on filters after 24 hours of incubation (average (standard deviation)), as illustrated in Figure 5. The presence of D. caveatum at 1/103 in mixtures inhibits the development at the aggregate stage. At 1/104 dilutions, the inhibition is no longer visible. The inhibition of D. rosarium is less significant at 24 hours than for other species, the predation of D. caveatum on this species will be reported elsewhere (R. J. Fitzhenry et al, in preparation). (0.01 MB PDF) [file pone.0000212.s001.pdf]

Table S1

	% of structures at the aggregate stage		
	Development	Mix 1-10^3	Mix 1-10^4
D. aureostipes	41 (31)	91 (8)	45 (10)
D. fasciculatum	16 (12)	71 (26)	51 (31)
D. mucoroides	59 (21)	88 (14)	58 (37)
D. rosarium	0 (0)	57 (43)	8 (7)
